# Editorial: Methods in molecular innate immunity: 2022

**DOI:** 10.3389/fimmu.2025.1576957

**Published:** 2025-02-28

**Authors:** Jörg H. Fritz, Thomas A. Kufer

**Affiliations:** ^1^ Department of Microbiology and Immunology, McGill University, Montréal, QC, Canada; ^2^ McGill University Research Center on Complex Traits (MRCCT), McGill University, Montréal, QC, Canada; ^3^ Dahdaleh Institute of Genomic Medicine (DIgM), McGill University, Montréal, QC, Canada; ^4^ Department of Immunology, Institute for Nutritional Medicine, University of Hohenheim, Stuttgart, Germany

**Keywords:** innate immunity, inflammation, methods, innate lymphoid cells, myeloid cells

Advances in immunology are inherently linked to progress in implementing novel methods as best illustrated by the development of the cre-lox technique that allows to analyse the effect of single genes on lymphocyte development and function by the generation of “conditional” knock-out mice (1). Development of novel as well as the optimization of existing technologies and methods furthers constant progress in biomedical research. The most recent game changer being the development of the bacterial immune system CRISPR-Cas9 into a universal tool for gene and genome editing (2).

Here in this Research Topic on “*Methods in Molecular Innate Immunity: 2022*” we provide a brief collection of state-of the art methods and protocols to enable in-depth studies of innate immune responses in *in vitro* cell culture systems as well as in *in vivo* models.

The identification of innate lymphoid cells (ILCs) and the rapid progress made in this field showed that ILCs exert essential roles in immune responses and tissue homeostasis (3). Four detailed protocols deal with the characterization of ILCs, their genetic manipulation, as well as the analysis of their metabolic states, respectively. Audouze-Chaud et al. provide a novel CRISPR/Cas9 protocol for efficient genetic knockout in human group 2 innate lymphoid cells (ILC2s) and discuss challenges and solutions. Sadeghalvad et al. present a detailed protocol for cytometric analysis of ILCs and provide tips for its successful implementation. Roy-Dorval et al. detail approaches for analysis of lipid uptake, storage, and fatty acid oxidation by ILC2s, while Krisna et al. provide a comprehensive framework for the immunometabolic analysis of primary murine ILC2s.

*In vivo* analysis of the distribution of immune cell subsets and their activation status was boosted by the development of single cell sequencing techniques, primarily single-cell RNA sequencing (4). Mindt et al. present a protocol to allow for spatial differentiation in single-cell RNA sequencing by using barcoded antibodies.

Macrophages and neutrophils are the first line of the innate immune defence. While macrophages emerged as key instruments to study innate immune responses due to their easy differentiation *in vitro* and their robustness in cell culture (5), neutrophils are extremely short-lived and isolation strategies for *in vitro* assays were only recently developed (6). In addition to its central role in host defence upon microbial challenge, the immune system is increasingly recognized as an integral part of fundamental physiological processes such as development, reproduction and wound healing, which involves a very close crosstalk with other body systems such as metabolism, the central nervous system and the cardiovascular system is evident (7). One prominent example being the discovery that TNFα is secreted from adipose tissue in obese mice and drives insulin resistance, highlighting that metabolic disorders are intimately linked to dysregulated immune responses (8). In an original research article, Iovino et al. present novel insights into the link of macrophage activation by saturated fatty acids and IRE1 RNase in metabolic reprogramming. Their work highlights a key role of IRE1α in HIF-1α-mediated glycolysis in macrophages independent of XBP1s.

Immune cell activation is tightly linked to changes in the metabolic wiring and mitochondrial activity. The development of devices to measure extracellular flux by redox potential changes in small volumes generated the basis to study cellular metabolic changes upon immune cell activation in great detail (9). Grudzinska et al. provide a protocol that exemplifies how extracellular flux (XF) analysis can be used to measure metabolism and oxidate burst in activated neutrophils.

The core function of innate immunity is the quick and often cell intrinsic reaction towards pathogen challenge (10). Zhi et al. detail investigations of the cGAS-STING signaling pathway and its modulation by traditional Chinese medicines. Furthermore, detailed studies of host-pathogen interactions at a time-resolved and molecular level are providing exiting new insights into the function of innate immune responses. Using a GFP fluorophore that is quenched when exposed to reactive oxygen species combined with a stable secondary fluorescent marker Hinman et al. provide a useful protocol to analyse killing of the human pathogen *Staphyococcus aureus* and neutrophil function in murine disease models.

We are living in an environment that is more and more polluted with chemicals that can affect the innate immune response. Plastic- and environment-derived bisphenol A (BPA) for example can accumulate in the human body and acts as an endocrine-disrupting compound. Dallio et al. analysed BPA levels in individuals and stimulated monocytes with BPA to assess metabolic and cytokine profiling.

This brief Research Topic will be helpful for research professional and trainees to implement novel methodologies to further detail the wealth of functions of the innate immune system upon microbial challenge and during inflammatory processes.
